# Do We Have the Evidence to Produce Tools to Enable the Identification and Personalization of Management of Women's Pelvic Floor Health Disorders Through the Perinatal and Perimenopausal Periods? ICI‐RS 2024

**DOI:** 10.1002/nau.70019

**Published:** 2025-02-23

**Authors:** Rohna Kearney, Stefano Salvatore, Vik Khullar, Christopher Chapple, Annika Taithongchai, Alan Uren, Paul Abrams, Alan Wein

**Affiliations:** ^1^ The Warrell Unit, Saint Mary's Hospital, Manchester University NHS Foundation Trust Manchester Academic Health Science Centre Manchester UK; ^2^ Institute of Human Development, Faculty of Medical & Human Sciences University of Manchester Manchester UK; ^3^ University Vita e Salute, IRCCS San Raffaele, Milan Milano Italy; ^4^ Department of Urogynaecology St Mary's Hospital, Imperial College London UK; ^5^ Sheffield Teaching Hospitals NHS Foundation Trust Sheffield UK; ^6^ Department of Urogynaecology King's College Hospital NHS Trust London UK; ^7^ Bristol Urological Institute, Southmead Hospital Bristol UK; ^8^ Desai Sethi Institute of Urology University of Miami Miller School of Medicine Miami Florida USA

**Keywords:** Pelvic floor dysfunction, Pelvic health personalisiation, perimenopausal health, Perinatal health, Tools

## Abstract

**Introduction:**

There is an increasing recognition of the impact of ageing on pelvic floor health and the consequences in populations with rising proportions of women over the age of 65 years. A think tank was held at the ICI‐RS 2024 to discuss the evidence to support the personalisation of women's pelvic floor health during the perinatal and perimenopausal period.

**Methods:**

Data was collected and presented on the evidence to support the development of tools to personalise pelvic floor health care. Epidemiological, imaging, patient‐reported outcomes, and evidence of tool development questionnaires were discussed. The current evidence and research gaps for potential intervention to prevent the pelvic floor disorders of pelvic organ prolapse, overactive bladder, urinary incontinence and faecal incontinence during the perinatal and perimenopasual time periods were discussed and identified.

**Results:**

Epidemiological studies highlight that vaginal delivery and in particular operative vaginal delivery is the single biggest modifiable risk factor for the future development of pelvic floor dysfunction. The oestrogen depletion resulting from the perimenopause and menopause can lead to the development of Genitourinary syndrome of menopause (GSM) which is associated with the risk of developing pelvic floor dysfunction. Ultrasound is a useful technique for assessing the pelvic floor and has been used to assess bladder neck mobility, distensibility of the puborectalis muscle and the striated urethral sphincter volume antenatally with some studies reporting a correlation between these measurements and the need for Caesarean section and development of postpartum stress urinary incontinence. Further studies are needed to standardise these measurements. There are no patient reported outcome questionnaires validated for use in the perinatal and postmenopausal period. The UR‐ choice tool has been developed to counsel women on the risk of postpartum pelvic floor disorders occurring. However, further evaluation in larger numbers is required.

**Conclusion:**

There is significant interest in developing tools to counsel women on the risks of developing pelvic floor dysfunction post partum and after the menopause. Further evaluation of the UR‐choice tool was considered a research priority. The timepoint of cervical screening for research into interventions such as pelvic floor health education, lifestyle optimisation and perimenopausal vaginal oestrogen supplementation was identified.

## 1 Introduction

Every country is experiencing growth in the size and proportion of the population over the age of 65 years. By 2030, 1 in 6 people in the world will be aged 60 or older [1]. The prevalence of pelvic floor health problems increases with age with significant physical, economic and socio‐psychological consequences [2]. In 2023 the Royal College of Obstetricians and Gynaecologists published its position statement on pelvic floor health which recommended expanding access to information and early interventions to improve pelvic floor health including a recommendation for maternity professionals to improve the identification of women at highest risk of pelvic floor dysfunction [3]. Childbirth is the single biggest modifiable risk factor for the development of pelvic floor dysfunction (PFD) and a time when many healthy women first engage with the healthcare system over a sustained period of time. The changes women experience around the menopause may accelerate the development of pelvic floor symptoms and there is increasing awareness of the importance of perimenopausal health. The perinatal and perimenopausal periods are windows of opportunity for interventions to reduce the lifetime burden of pelvic floor dysfunction.

The Health Foundation has defined person‐centred care as a framework with four main principles; affording people compassion, dignity and respect, offering coordinated care, support or treatment, offering personalised care and enabling people to recognize and develop their own strengths and abilities so they can live an independent life [4]. Shared decision making is an integral part of personalised care and it is the responsibility of health care professionals to work with patients to codevelop high quality evidence‐based tools to support and enable informed personalised healthcare decisions. There is a need to develop tools that can enable the identification of women who are at high risk of developing PFD after childbirth and the menopause. This would allow the opportunity to study the impact of targeted interventions such as elective caesarean delivery, pelvic floor muscle training, weight loss interventions and vaginal oestrogen therapy.

The ICI‐RS group in June 2024 discussed the evidence available to produce tools to enable the personalisation of pelvic floor health in the perinatal and perimenopausal period. The discussion focused on evidence from epidemiological, imaging and questionnaire‐based studies. Common pelvic floor disorders such as pelvic organ prolapse, urinary and faecal incontinence were considered in the literature reviews and presentations. This paper outlines the evidence reviews that supported this discussion, the discussion that took place and the research questions that were developed as a basis for future research proposals.

## 2 Epidemiology Studies

**Figure 1:**
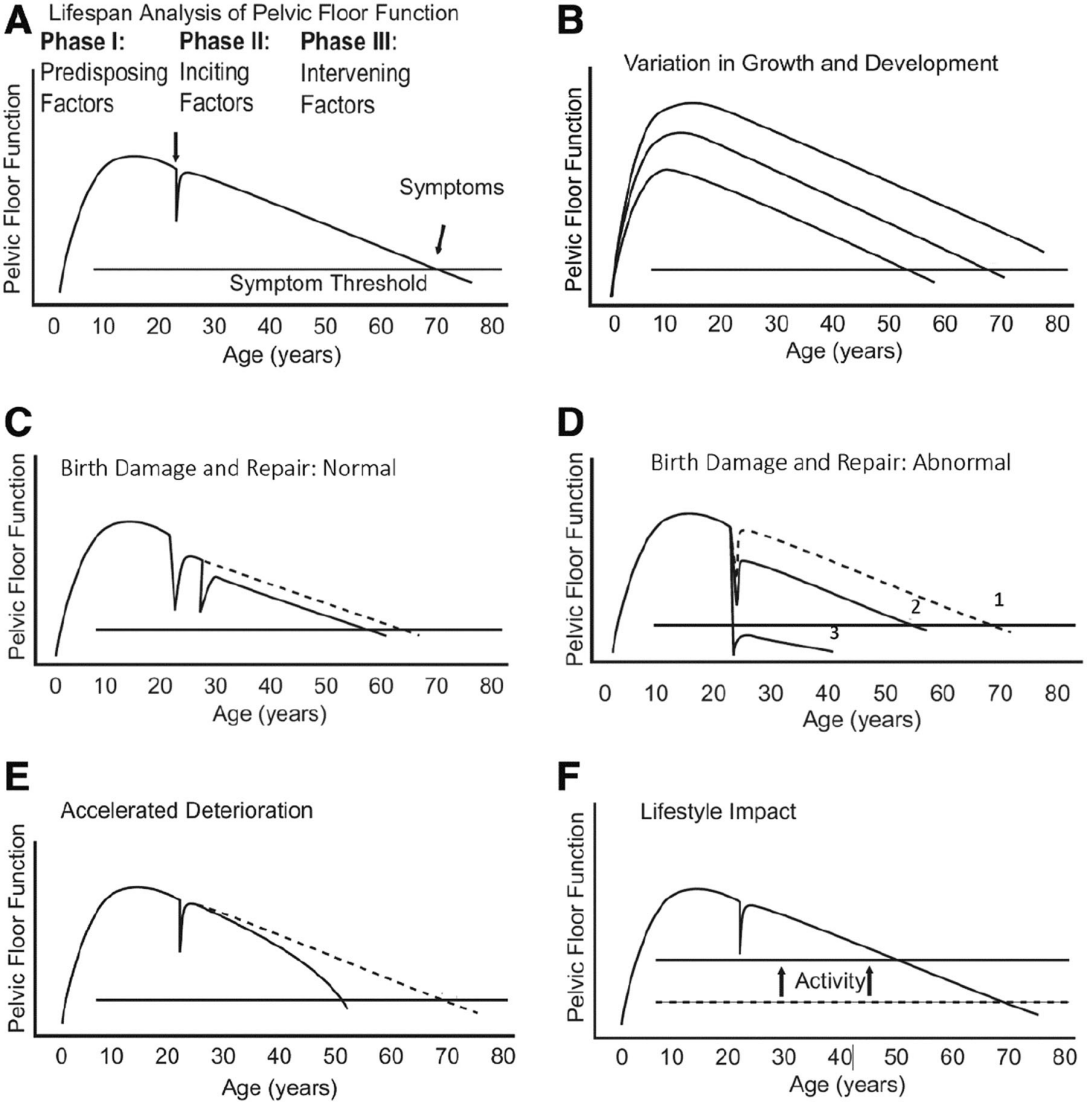
Pelvic floor injury during vaginal birth is life‐altering and preventable: what can we do about it? Reprinted with permission from J. O. L. DeLancey et al., American Journal of Obstetrics & Gynecology, 2024, Volume 230, Issue 3, 279‐294.e2. © 2023 Elsevier Inc.

Pelvic Floor Dysfunction can occur any time in a woman's life, although the prevalence of symptoms increases with age. In a lifespan model DeLancey et al [5] described the importance of predisposing, inciting and intervening causal factors of PFDs (Figure 1). Birth injury, ageing, lifestyle factors and activity can contribute to women developing symptoms of pelvic floor dysfunction such as pelvic organ prolapse, urinary and faecal incontinence.

Pregnancy and childbirth have always been related to the development of PFD with mode of delivery and vaginal birth in particular consistently reported as one of the most important inciting causal factors. This has been confirmed by Blomquist et al who followed up women after their first delivery and looked at the incidence of PFDs in relation to mode of delivery after one or two decades [6]. The cumulative incidence since delivery of stress urinary incontinence, overactive bladder, anal incontinence and pelvic organ prolapse was more positively correlated with operative vaginal delivery and spontaneous vaginal delivery compared to caesarean section.

**Figure 2:**
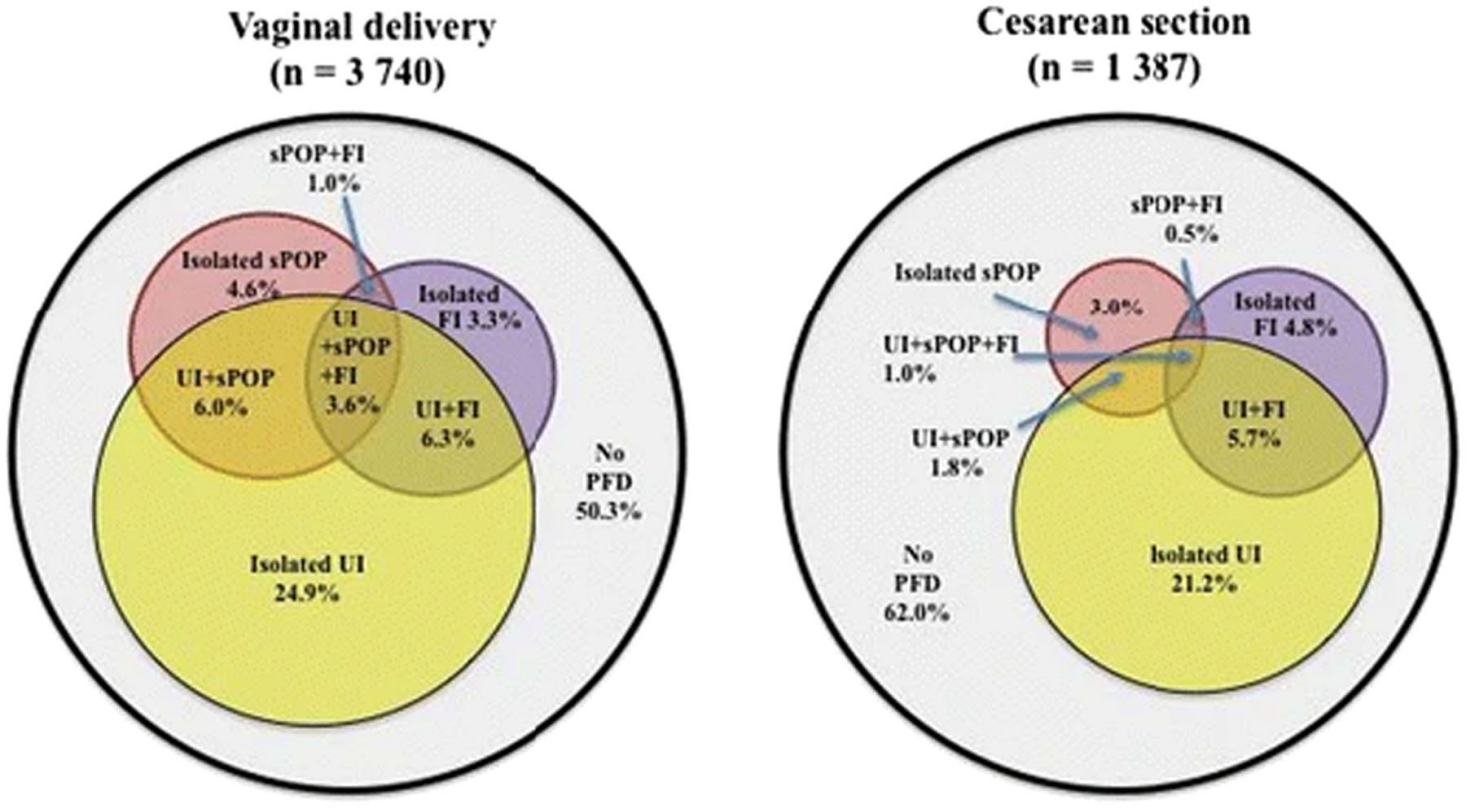
Clustering of pelvic floor disorders 20 years after one vaginal or one caesarean birth. Reprinted with permission from M. Gyhagen et al., International Urogynecology Journal, 2015, Volume 26, Issue 8, 1115‐1121. © 2015 Springer Nature.

A large registry based national cohort study of primipara who delivered one child by either vaginal delivery or caesarean section with no further births reported a prevalence of 46.5% for any PFD symptom 20 years later [7]. Two or more PFD symptoms occurred in 14.8% and doubled after vaginal delivery (17.1%) compared with caesarean section (8.4%) with the strongest association between vaginal delivery and having all three symptoms; prolapse, urinary and anal incontinence (OR 5.2) (Figure 2). Another prospective cohort study of 670 nulliparous women from early pregnancy to 1 year postpartum reported that the presence of stress urinary incontinence during pregnancy increased the risk of stress urinary incontinence (SUI) symptoms at 1‐year postpartum (RR2.48) [8]. Urgency incontinence (UUI) during pregnancy also increased the risk of persistent urgency incontinence postpartum (RR4.07). Vaginal delivery increased the risk of SUI postpartum but not UUI (RR 2.63).

Other contributing factors to the development of PFD other than mode of delivery should be considered and in the last few years, researchers have focused their efforts on trying to identify the modifiable and the inevitable ones. In 2021 The National Institute of Clinical Excellence in the UK produced a guideline (NG210) on the prevention and nonsurgical management of PFDs [9]. They reviewed the available literature and classified risk factors as obstetric or non‐obstetric (Table 1). Non‐obstetric modifiable risk factors in this guideline included a BMI over 25 kg/m^2^, smoking, lack of exercise, constipation and diabetes. Obstetric risk factors were related to pregnancy (maternal age > 30 years and multiparity) and related to labour (operative vaginal delivery, occipito‐posterior position, second stage of labour longer than 1 h and anal sphincter injury). Non‐obstetric risk factors from other studies include age, family history of PFD, ethnicity, previous hysterectomy, obesity, irritable bowel syndrome, dementia, diabetes, neurological illness such as multiple sclerosis, Parkinson's disease and recurrent UTIs (Table 2) [10]. Epidemiological studies have demonstrated ethnic differences in the occurrence of pelvic floor injury

**Table 1:** Risk factors for pelvic floor dysfunction identified in NICE NG 210 [9].

**Modifiable risk factors** A body mass index (BMI) over 25 kg/m^2^ Smoking Lack of exercise Constipation Diabetes **Non‐modifiable risk factors** Age (risk increases with increasing age) Family history of urinary incontinence, overactive bladder or faecal incontinence Gynaecological cancer and any treatments for this Gynaecological surgery (such as a hysterectomy) Fibromyalgia Chronic respiratory disease and cough (chronic cough may increase the risk of faecal incontinence and flatus incontinence) **Related to pregnancy** Being over 30 years when having a baby Having given birth before their current pregnancy **Related to labour** Assisted vaginal birth (forceps or vacuum) A vaginal birth when the baby is lying face up (occipito‐posterior) An active second stage of labour taking more than 1 h Injury to the anal sphincter during birth

**Table 2:** Additional risk factors for pelvic floor dysfunction factors discussed at ICI‐RS 2024.

Ethnicity Irritable Bowel Syndrome Neurological disease Recurrent UTIs Urethral sphincter volume on ultrasound less than 1.4cc Antenatal stress urinary incontinence Antenatal urge urinary incontinenceAntenatal bladder neck movement on ultrasound greater than 1 cm or 30 degrees Maternal height < 160 cm Foetal birth weight > 4 Kg

and types of pelvic floor dysfunction. Urgency urinary incontinence is more prevalent in black women and stress urinary incontinence in white women [11]. Asian women are more likely to sustain an obstetric anal sphincter injury than white women [12].

In general, PFDs have a prevalence that increases with age, and this can have multifactorial reasons such as the reduction/cessation of oestrogen production in the perimenopausal period, the ageing process per sé and comorbidities. Menopause is certainly the most important factor because the genital and the lower urinary tract have oestrogen receptors. The lack of oestrogens correlates with histological and functional changes and the onset of genital and urinary symptoms. For this reason, the term Genitourinary Syndrome of Menopause (GSM) has been proposed. Genitourinary syndrome of menopause (GSM) is a term that describes the multiple changes occurring in the external genitalia, pelvic floor tissues, bladder and urethra, and the sexual sequelae of loss of sexual function and libido, caused by hypoestrogenism during the menopause transition and postmenopause [13]. The prevalence of GSM is 4% during perimenopause, rising after menopause to 25% after 1 year and to 47% after 3 years [14].

The importance of oestrogen depletion in the occurrence of pelvic floor dysfunction has been described by Erekson et al who showed a correlation of vulvovaginal symptoms with PFD in postmenopausal women [15]. When these women were compared to a group of women without vulvovaginal symptoms, the authors showed that all urinary symptoms (urinary stress incontinence, overactive bladder and dysuria), anal incontinence and pelvic organ prolapse were significantly more common in women with associated vulvovaginal symptoms. Moreover, the severity of GSM symptoms is worse in women who had iatrogenic causes (surgical, radiotherapy etc) compared to spontaneous menopause.

## 3 Imaging Studies

Ultrasound is a useful noninvasive method of visualising the anatomy of the lower urinary tract either as a static or dynamic technique. Interpretation of ultrasound studies is influenced by parity and ethnicity. Findings may differ if the individuals studied are nulliparous, parous or pregnant [16, 17, 18, 19]. There also are racial differences in urethral sphincter volume with black women having larger rhabdosphincter volumes than white women which may explain differences in urinary symptoms after vaginal delivery [20]. It is important when interpreting the measurements or diagnostic parameters of ultrasound studies that both techniques, particularly for dynamic studies and the individuals being studied are comparable.

Transperineal ultrasound is a technique where the ultrasound probe is applied to the perineum and the urethra and bladder are visualised usually in the supine position, but a seated device has also been reported which may allow easier ultrasound assessment of prolapse [21]. The pelvic organs and in particular bladder neck mobility can be visualised at rest and at Valsalva. The technique for increasing intra‐abdominal pressure should be standardised. Due to the external application of the ultrasound probe, this technique is useful for imaging in pregnancy. Bladder neck mobility has been assessed in nonpregnant, asymptomatic, nulliparous women and ranges for coughing and Valsalva have been reported [16, 22]. However, there are a number of problems as the techniques used to produce bladder neck mobility have varied between a fixed Valsalva pressure to maximum straining without a specified instruction. The instruction to produce the strain given varies the amount of intra‐abdominal pressure as well as whether the pelvic floor contracts or relaxes. The number of times the Valsalva is attempted alters the degree of bladder neck movement [23, 24]. There are eight studies which have assessed bladder neck movement in pregnancy [19, 25, 26, 27, 28, 29, 30, 31, 32, 33]. The studies where bladder neck movement was assessed before 20 weeks showed less movement than those carried out after 28 weeks. Importantly those studies assessing bladder neck mobility after 28 weeks correlated with post‐partum stress urinary incontinence. King and Freeman used a standardised method of Valsalva and assessed antenatal bladder neck movement greater than 1 cm or 30 degrees as being associated with an increased risk of post‐partum stress incontinence with a RR of 8.7 which was superior to the symptom of antenatal stress urinary incontinence predicting post‐natal urinary incontinence with a RR of 3.3 [19]. A subsequent randomised study showed that selecting nulliparous women who have antenatal bladder neck hypermobility and given supervised physiotherapy by a physiotherapist reduced the rate of post‐partum stress urinary incontinence to 19.2% compared with 32.7% in the control group who received verbal instructions on pelvic floor exercises alone (RR 0.59 [0.37‐0.92]) [34].

Three‐dimensional ultrasound can be used to measure the urethral sphincter volume. This is a reproducible measurement and a urethral striated sphincter volume less than 1.4cc is associated with urodynamics stress incontinence as well as predicting less good outcome of continence surgery [35, 36, 37]. Toozs‐Hobson showed that the sphincter volume decreased with vaginal delivery by 10% on average and if emergency Caesarean section was carried out after 5 cm cervical dilation. This suggests the data on comparing the pelvic floor outcomes and delivery method need to separate elective from emergency Caesarean section [31].

Assessment of the pelvic floor and levator ani hiatus has been carried out antenatally and postnatally with MRI and 3D ultrasound [38, 39, 40, 41]. These techniques do not predict the development of incontinence or prolapse. However, some studies suggest that the levator ani hiatus which can be measured with 2D, and 3D ultrasound may predict the need for emergency Caesarean section in labour with one study showing that women who delivered vaginally had more distensible puborectalis muscles measured by ultrasound at 32 weeks compared to women who delivered by caesarean section [42, 43]. A larger study is needed to further evaluate the role of antenatal puborectalis distensibility in the prediction of vaginal delivery and whether this correlates with future risk of pelvic floor dysfunction.

## 4 Patient‐Reported Assessment Questionnaires

Although many patient‐reported outcome measures (PROMs) have been developed to evaluate pelvic health for use in urogynaecology populations [44], few have been specifically validated for use in the perinatal period [45]. In 2017 the International Consortium for Health Outcomes Measurement (ICHOM) group devised a standardised outcome set, that includes a series of PROMs/PREMs to evaluate care during pregnancy and up to 6 months post‐partum [46] These have been used so far mostly in a research capacity, but also in a clinical context [47]. However, recent systematic reviews have highlighted the limitations of existing PROMs in evaluating post‐partum recovery and the need for the robust development of a new and comprehensive tool [45, 48]. Currently, few tools specifically assess patient‐reported PFDs in pregnancy and post‐partum in a complete and integrated way. One has been developed in the German language [49] and was based on the widely used Australian Pelvic Floor Questionnaire [50]. A recent development in the English language is the Perinatal Pelvic Health Self‐Assessment Questionnaire (PPSAQ) [51]. The aim is to enable women to self‐report risk factors and monitor PFD symptoms throughout the perinatal period, and to inform appropriate referrals to new pelvic health centres that are established in England. This questionnaire has been developed alongside the existing modular International Consultation in Incontinence Questionnaires (ICIQ) [52]. The availability of self‐completed questionnaires validated in the perinatal population shows promise, however, the integration of PROMs/PREMs in‐network healthcare systems such as perinatal care has many complexities, given the intricate patient pathway and appointment schedules. Challenges remain to the provision of information technology infrastructures that integrate with the local healthcare systems, including solutions to cultural, literacy and language barriers to ensure tools remain accessible to completion by all [47].

Although there are questionnaires available specifically for the menopausal population, currently assessment centres around broader health‐related quality of life domains such as physical, emotional, and social functioning [53]. The assessment of pelvic floor health in menopausal women is under‐researched and no population‐specific tools have been developed for this patient group. However, it is recommended by the ICI that existing recommended questionnaires should be used, if possible, as this standardises use and avoids the proliferation of new questionnaires [54].

## 5 Perinatal Period

Large cohort studies contribute to our understanding of the development of PFD following delivery. One of the largest was the SWEPOP study, a national cohort study carried out in Sweden of women who delivered in the 1980s (*n* = 5236 women who had a single vaginal delivery or a single caesarean delivery) [55]. They showed the greatest risk factors for developing symptomatic pelvic organ prolapse (POP) were vaginal delivery (OR 2.55 (95% CI 1.98–3.28)) and maternal height of less than 160 cm with a foetal birthweight greater than 4 kg (OR 2.06 (95% CI 1.19–3.55)). The same year, the ProLong study group published their 12‐year findings, a longitudinal study assessing women at 3 months, 6 years and 12 years following delivery, carried out in Scotland, England and New Zealand [56]. The most significant risk factor for development of faecal incontinence was a single forceps delivery (OR 2.08 (95% CI 1.53–2.85)) as well as raised BMI (OR 1.52 (95% CI 1.06–2.17)). Fu et al also found similar risk factors, but in addition found urinary incontinence during pregnancy (OR 4.42), constipation (OR 2.46) and bi‐parietal diameter at 32 weeks (OR 51.67) to increase risks for development of post‐partum pelvic floor dysfunction [57].

With regard to the link between forceps delivery and pelvic floor dysfunction, this has been well established. We know there is a clear association between forceps and levator ani avulsions (OR 4.35 (95% CI 2.56–7.40)) and those with levator ani avulsions (OR 2.28 (95% CI 1.34–3.91)) and greater hiatal area (OR 3.28 (95% CI 1.96–5.50)) are at greatest risk of developing symptomatic POP, demonstrated in a cross‐sectional study of 850 women 20 years after their first delivery [58].

The detection of nerve injury related to delivery is more difficult to diagnose. EMG studies of the external anal sphincter and postpartum pudendal terminal motor latency studies showed that multiparity, forceps delivery, increased length of second stage, anal sphincter injury and high birth weight were important factors linked to pudendal nerve damage [59]. Quantitative nerve testing has shown that mode of birth has a significant impact on sensation with caesarean delivery being neuroprotective and vaginal delivery showing a transient effect but operative delivery being associated with more prolonged neurological impairment [60].

Some risk factors for PFD are unmodifiable, the factors standing out as amenable to change are mode of birth and the use of forceps. In labour, if urgent delivery or assistance is required during the second stage the options include ventouse, forceps or caesarean section. Delivery by ventouse is not always feasible and caesarean section at full dilation has significant maternal morbidity and implications for mother and babies in future pregnancy, so forceps delivery is often considered. It is imperative to ensure forceps are used appropriately and only when required and training continues to optimise ventouse use. Women at high risk for development of pelvic floor dysfunction should be offered a planned caesarean section, if they wish, once they have considered the individualised risks presented to them including the impact on future childbearing. Each woman will have a different threshold for deciding whether the risks for developing pelvic floor disorders are high enough to consider caesarean birth and this should be an informed individual choice.

## 6 Perimenopausal Period

The menopause has a significant impact on pelvic floor function, and it is well‐recognised that the consequences of age‐related pelvic floor weakness combined with a reduction in oestrogen are important factors. It has been known for many years that a substantial proportion of incontinence‐free post‐menopausal women develop urinary incontinence later in life. A prospective study of women over 2 years recruited aged 55–75 years showed that among the 345 women without any incontinence at baseline 19% reported some incontinence at 1 and 2 years. Independent predictors of incontinence included white race, use of vaginal oestrogen cream, vaginal dryness, vaginal discharge, 6 or more lifetime urinary tract infections, vaginal colonisation with E. coli and diabetic peripheral neuropathy. A history of hysterectomy and any vaginal symptoms were also strong predictors (OR 1.8, 1.7) [61].

A recent Swedish study based on a national cohort of nulliparous women aged 25–64 years (*N* = 624,049) presented detailed descriptive measures of accidental leakage of liquid or solid stool and gas and identified abnormal stool consistency with gas and liquid stool as the strongest risk factor for accidental bowel leakage. The low rates of isolated leakage of solid stool were felt to support the impression that dysfunction of the continence mechanism of the pelvic floor had a negligible role in bowel incontinence. This study provides important data that can be considered for comparisons with parous women who may have birth‐related injuries [62].

In addition to the pre‐existing obstetric‐related risk factors, other medical conditions such as diabetes and obesity and certain occupations such as cleaning or nursing have been highlighted as significant for some women in developing urinary or faecal incontinence or pelvic organ prolapse [63, 64] The additional risk factors in postmenopausal women have not been investigated as extensively but the perimenopause appears to be a moment in a woman's life that intervention for or modification of risk factors could be introduced.

## 7 Tools

There has been enormous interest in the development of models to predict outcomes relating to pelvic floor disorders. This work will potentially allow the identification of women at higher risk of developing urinary or faecal incontinence and allow preventative strategies to be applied, examples being pelvic floor muscle training, weight control, and the choice of an elective caesarean section rather than vaginal delivery, which might help improve their outcome. A study looking at the possibility of developing a predictive model looked at variables from 2 previous study cohorts of 20,000 women, half each from the ProLong and SwePOP studies and reported that models were able to discriminate between women who experienced bothersome symptoms or had received treatment at 12 and 20 years. Route of delivery and family history were strong predictors of each PFD. Urinary incontinence before and during index pregnancy was a strong predictor for the development of all PFDs in most models 12 years after delivery. Further refinement and external validation of these models are required [65].

Using risk factors identified before and during childbirth, prognostic models have been also developed to predict the risk of developing urinary incontinence, faecal incontinence and PFDs [65]. The UR‐choice tool was developed as a scoring tool to counsel women antenatally on the risk of developing PFD after vaginal delivery based on risk factors [66]. No prognostic tools have been developed to predict the onset of PFD in the peri or postmenopausal period.

## 8 Conclusion

There have been few studies investigating interventions to address modifiable risk factors to prevent the onset of pelvic floor dysfunction. The NICE guideline on prevention of pelvic floor dysfunction identified 7 studies of lifestyle interventions and 15 studies of pelvic floor muscle training to prevent pelvic floor dysfunction [9]. Six studies assessed the effectiveness of lifestyle changes on the development of urinary incontinence and one study assessed fibre intake and the development of faecal incontinence. All lifestyle intervention studies were considered to provide very low‐quality evidence. Of the 15 studies assessing PFMT 6 recruited antenatal women and 7 postnatal women. The 2 community‐based studies provided low‐quality conflicting evidence of the benefit of PFMT in reducing urinary symptoms. Obstetric studies showed low to moderate‐quality evidence of effect of PFMT in reducing urinary incontinence and pelvic organ prolapse in the short term postpartum.

In the UK, the Women's Health Strategy published in 2022 encourages the expansion of women's health hubs around the country and other models of ‘one‐stop clinics’, bringing essential women's services together to support women to maintain good health and create efficiencies for the NHS [67]. The women's health hubs cover a range of conditions but pelvic floor health is considered an optional area for development. Integrating pelvic floor health into reproductive and gynaecology care in women's health hubs and maternity care will improve access to preventative treatment such as pelvic floor muscle training, encourage optimisation of pelvic floor health before pregnancy and promote better pelvic floor health in the peri and postmenopausal period.

There was a wide consensus that further validation of the UR choice tool would be helpful and that it would be important to involve women in this evaluation. Recently developed PROMs validated specifically for the self‐reporting of PFDs and risk factors within the perinatal period show promise if successfully integrated into clinical practice. Whilst there is growing awareness of the impact of birth pelvic floor trauma, it is not routinely discussed by midwives and obstetricians. This means that women experiencing stress urinary incontinence antenatally are not always identified and referred for PFMT. The benefits of interventions such as PMFT need to be better evaluated in this context. Personalised risk calculators should differentiate between the benefits of elective and emergency caesarean delivery. It is recognized that although forceps delivery is a significant risk factor for the development of PFD, caesarean delivery at full dilatation is also associated with morbidity and long‐term consequences for future deliveries.

Research should focus on what women want to know in advance of pregnancy and antenatally and when and how they want to receive information on their personalised risk of pelvic floor dysfunction. It was recognised that women do not always report symptoms and there was a need for research on the best way to elicit them.

Regarding the perimenopausal period, it was considered that the 3 yearly cervical smear screening provided an opportunity to discuss pelvic floor health before the menopause but qualitative research is needed to understand when and how women would like to discuss their pelvic floor health. The availability of oestradiol vaginal preparations over the counter in the UK increases access for women with symptoms of GSM and research on whether starting this before onset of GSM was considered relevant. Providing women with information was considered very important to educate on common pelvic floor symptoms and advise on lifestyle treatments, interventions and where to access help. The age of 45‐50 was considered a critical period for this. The paucity of data on long‐term use of vaginal oestrogens was discussed and the fact that the enclosed safety information is the same as that of the combined oral contraceptive pill which contains much higher dose of oestrogen overstates the risks of potential side effects and complications. Risk of drug interaction with other medications is also unclear.

## 9 Research Questions

What is the optimum gestation to assess bladder neck movement on ultrasound in pregnancy?

∘ Further longitudinal study is required.

Can antenatal 3D ultrasound of urethral sphincter volume predict post‐natal stress urinary incontinence?

∘ Larger cohort study measuring volume and symptoms antenatally and postnatally is required.

Is it possible to standardise the method of increasing abdominal pressure for ultrasound assessment of bladder neck and urethral mobility?

∘ Feasibility study required to determine reproducibility and reliability of methods to increase intrabdominal pressure.

What are the predictors for pelvic organ prolapse, urinary and faecal incontinence, both postnatally and in menopause?

∘ Larger epidemiological studies are required.

What is the core outcome set for evaluating pelvic floor symptoms in postnatal and perimenopausal women?

∘ Codesign and evaluation studies of questionnaires and other tools investigating symptoms are required.

Is it necessary to establish a pelvic floor condition‐specific PROM for women in the perimenopause?

∘ A qualitative study with service users to explore their needs regarding perimenopausal health is required.

Can UR choice tool be used routinely to advise women of the risk of pelvic floor dysfunction associated with delivery and is it acceptable to women?

∘ A longitudinal cohort study evaluating the UR choice tool is required.

How and when do women wish to receive information on PFD?

∘ A qualitative study exploring women's needs and wishes regarding information of PFD is required.

Is there a role for vaginal oestrogen treatment in asymptomatic women perimenopausally to prevent the onset of GSM and PFD?

∘ An RCT to assess the effectiveness of vaginal oestrogen to prevent the onset of GSM and PFD is required.

What is the long‐term safety of vaginal oestrogen in women with contraindications to systemic oestrogen?

∘ A prospective study of women using vaginal oestrogen is required.

What is the risk of drug interactions with vaginal oestrogen and other medications such as warfarin?

## Author Contributions

All Authors have made substantial contributions to the conception or design of the work, or the acquisition, analysis, or interpretation of data for the work. Drafting the work or reviewing it critically for important intellectual content. Final approval of the version to be published. Agreement to be accountable for all aspects of the work in ensuring that questions related to the accuracy or integrity of any part of the work are appropriately investigated and resolved.

## Ethics Statement

The authors have nothing to report.

## Conflicts of Interest

The authors declare no conflicts of interest.

## Data Availability

Data sharing is not applicable to this article, as no new data were created or analyzed in this study.

## References

[1] World Health Organization, Ageing and Health (WHO, 2024) , https://www.who.int/news-room/fact-sheets/detail/ageing-and-health.

[2] R. A. Peinado Molina , A. Hernández Martínez , S. Martínez Vázquez , and J. M. Martínez Galiano , “Influence of Pelvic Floor Disorders on Quality of Life in Women,” Frontiers in Public Health 11 (2023): 1180907, 10.3389/fpubh.2023.1180907. 37942254 PMC10629477

[3] RCOG Policy Position: Pelvic Floor Health, https://www.rcog.org.uk/about-us/campaigning-and-opinions/position-statements/pelvic-floor-health-position-statement/.

[4] Person‐Centred Care Made Simple. The Health Foundation 2014, https://www.health.org.uk/publications/person-centred-care-made-simple.

[5] J. O. L. DeLancey , M. Masteling , F. Pipitone , J. LaCross , S. Mastrovito , J. A. Ashton‐Miller , “Pelvic Floor Injury During Vaginal Birth Is Life-Altering and Preventably: What We can do About It?,” American Journal of Obstetrics & Gynecology 230, no. 3 (2024): 279–294.e2. 38168908 10.1016/j.ajog.2023.11.1253 PMC11177602

[6] J. L. Blomquist , A. Muñoz , M. Carroll , and V. L. Handa , “Association of Delivery Mode With Pelvic Floor Disorders After Childbirth,” Journal of the American Medical Association 320, no. 23 (2018): 2438–2447, 10.1001/jama.2018.18315. 30561480 PMC6583632

[7] M. Gyhagen , S. Åkervall , and I. Milsom , “Clustering of Pelvic Floor Disorders 20 Years After One Vaginal or One Cesarean Birth,” International Urogynecology Journal 26, no. 8 (August 2015): 1115–1121, 10.1007/s00192-015-2663-3. 25708677

[8] M. H. Jansson , K. Franzén , G. Tegerstedt , A. Hiyoshi , and K. Nilsson , “Stress and Urgency Urinary Incontinence One Year After a First Birth‐Prevalence and Risk Factors. A Prospective Cohort Study,” Acta Obstetricia et Gynecologica Scandinavica 100, no. 12 (2021): 2193–2201, 10.1111/aogs.14275. 34699060

[9] National Guideline Alliance (UK) , Prediction Tools for Pelvic Floor Dysfunction: Pelvic Floor Dysfunction: Prevention and Non‐Surgical Management: Evidence Review D (London: National Institute for Health and Care Excellence (NICE), 2021). 35438866

[10] D. Altman , R. Cartwright , M. C. Lapitan , et al., “Epidemiology of Urinary Incontinence (UI) and Other Lower Urinary Tract Symptoms (LUTS), Pelvic Organ Prolapse (POP) and Anal Incontinence (AI).” in Incontinence: 6th International Consultation on Incontinence, Tokyo, September 2016, eds. P. Abrams , L. Cardozo , A. Wagg , A. J. Wein (Bristol: International Continence Society, 2017), 1–141.

[11] Jan C. L. G. Sears , J. Wright , J. O'Brien , et al., “The Racial Distribution of Female Pelvic Floor Disorders in an Equal Access Health Care System,” Journal of Urology 181, no. 1 (2009): 187–192, 10.1016/j.juro.2008.09.035. 19013607

[12] I. Gurol‐Urganci , D. Cromwell , L. Edozien , et al., “Third‐ and Fourth‐Degree Perineal Tears Among Primiparous Women in England Between 2000 and 2012: Time Trends and Risk Factors,” BJOG: An International Journal of Obstetrics & Gynaecology 120 (2013): 1516–1525. 23834484 10.1111/1471-0528.12363

[13] E. M. Farrell , “Genitourinary Syndrome of the Menopause,” Aus Fam Phy 46 (2017): 481–484. 28697291

[14] L. Dennerstein , E. C. Dudley , J. L. Hopper , J. R. Guthrie , and H. G. Burger , “A Prospective Population‐Based Study of Menopausal Symptoms,” Obstetrics and Gynecology 96, no. 3 (2000): 351–358. 10960625 10.1016/s0029-7844(00)00930-3

[15] E. A. Erekson , F. Y. Li , D. K. Martin , and T. R. Fried , “Vulvovaginal Symptoms Prevalence in Postmenopausal Women and Relationship to Other Menopausal Symptoms and Pelvic Floor Disorders,” Menopause 23, no. 4 (April 2016): 368–375, 10.1097/GME.0000000000000549. 26645820 PMC4814326

[16] H. Reed , R. M. Freeman , A. Waterfield , and O. Adekanmi , “Prevalence of Bladder Neck Mobility in Asymptomatic Non‐Pregnant Nulliparous Volunteers,” BJOG: An International Journal of Obstetrics & Gynaecology 111 (2004): 172–175. 14723757 10.1046/j.1471-0528.2003.00043.x-i1

[17] C. R. Chapple , C. W. Helm , S. Blease , E. J. G. Milroy , D. Rickards , and J. L. Osborne , “Asymptomatic Bladder Neck Incompetence in Nulliparous Females,” British Journal of Urology 64 (1989): 357–359. 2819385 10.1111/j.1464-410x.1989.tb06042.x

[18] R. Karmarkar , A. Digesu , R. Fernando , and V. Khullar , “Ultrasound Assessment of Urethral Structure and Bladder Neck Position in Women With Different Parities,” International Urogynecology Journal 33 (2022): 613–618. 33660002 10.1007/s00192-021-04715-z

[19] J. K. King and R. M. Freeman , “Is Antenatal Bladder Neck Mobility a Risk Factor for Postpartum Stress Incontinence?,” BJOG: An International Journal of Obstetrics & Gynaecology 105 (1998): 1300–1307. 10.1111/j.1471-0528.1998.tb10009.x 9883922

[20] A. Derpapas , S. Ahmed , G. Vijaya , et al., “Racial Differences in Female Urethral Morphology and Levator Hiatal Dimensions: An Ultrasound Study,” Neurourology and Urodynamics 31 (2012): 502–507. 22190140 10.1002/nau.21181

[21] G. N. Schaer , R. Siegwart , D. Perucchini , J. O. DeLancey , “Examination of Voiding in Seated Women Using a Remote‐Controlled Ultrasound Probe,” Obstetrics and Gynecology 91 (1998): 297–301. 9469294 10.1016/s0029-7844(97)00681-9

[22] U. M. Peschers , G. Fanger , G. N. Schaer , et al., “Bladder Neck Mobility in Continent Nulliparous Women,” British Journal of Obstetrics and Gynaecology 108 (2001): 320–324. 11281475 10.1111/j.1471-0528.2001.00066.x

[23] J. A. Tumbarello , Y. Hsu , C. Lewicky‐Gaupp , S. Rohrer , and J. O. L. DeLancey , “Do Repetitive Valsalva Maneuvers Change Maximum Prolapse on Dynamic Mri?,” International Urogynecology Journal 21 (2010): 1247–1251. 20544342 10.1007/s00192-010-1178-1 PMC2932797

[24] D. Howard , J. M. Miller , J. O. L. Delancey , J. A. Ashton‐Miller , and D. Howard , “Differential Effects of Cough, Valsalva, and Continence Status on Vesical Neck Movement,” Obstetrics & Gynecology 95 (2000): 535–540. 10725485 10.1016/s0029-7844(99)00618-3 PMC1226414

[25] U. Peschers , G. Schaer , C. Anthuber , J. Oldelancey , and B. Schuessler , “Changes in Vesical Neck Mobility Following Vaginal Delivery,” Obstetrics & Gynecology 88 (1996): 1001–1006. 8942842 10.1016/s0029-7844(96)00338-9

[26] S. Meyer , A. Schreyer , P. De Grandi , and P. Hohlfeld , “The Effects of Birth on Urinary Continence Mechanisms and Other Pelvic‐Floor Characteristics,” Obstetrics and Gynecology 92 (1998): 613–618. 9764638 10.1016/s0029-7844(98)00248-8

[27] S. Meyer , O. Bachelard , and P. De Grandi , “Do Bladder Neck Mobility and Urethral Sphincter Function Differ During Pregnancy Compared With During the Non‐Pregnant State?,” International Urogynecology Journal 9 (1998): 397–403. 10.1007/BF02199575 9891962

[28] H. P. Dietz and M. J. Bennett , “The Effect of Childbirth on Pelvic Organ Mobility,” Obstetrics & Gynecology 102 (2003): 223–228. 12907092 10.1016/s0029-7844(03)00476-9

[29] J. Wijma , A. E. W. Potters , B. T. H. M. de Wolf , et al., “Anatomical and Functional Changes in the Lower Urinary Tract During Pregnancy,” BJOG: An International Journal of Obstetrics & Gynaecology 108 (2001): 726–732. 11467699 10.1111/j.1471-0528.2001.00123.x

[30] J. Wijma , A. E. W. Potters , B. T. H. M. de Wolf , D. J. Tinga , and J. G. Aarnoudse , “Anatomical and Functional Changes in the Lower Urinary Tract Following Spontaneous Vaginal Delivery,” BJOG: An International Journal of Obstetrics & Gynaecology 110 (2003): 658–663. 12842056

[31] P. Toozs‐Hobson , J. Balmforth , L. Cardozo , V. Khullar , and S. Athanasiou , “The Effect of Mode of Delivery on Pelvic Floor Functional Anatomy,” International Urogynecology Journal 19 (2008): 407–416. 10.1007/s00192-007-0455-0 17896066

[32] J. Stær‐Jensen , F. Siafarikas , G. Hilde , J. Š. Benth , K. Bø , and M. E. Engh , “Postpartum Recovery of Levator Hiatus and Bladder Neck Mobility in Relation to Pregnancy,” Obstetrics & Gynecology 125 (2015): 531–539. 25730212 10.1097/AOG.0000000000000645

[33] K. Jundt , I. Scheer , B. Schiessl , K. Karl , K. Friese , and U. Peschers , “Incontinence, Bladder Neck Mobility, and Sphincter Ruptures in Primiparous Women,” European Journal of Medical Research 15 (2010): 246–252. 20696633 10.1186/2047-783X-15-6-246 PMC3351993

[34] E. T. C. Reilly , R. M. Freeman , M. R. Waterfield , A. E. Waterfield , P. Steggles , and F. Pedlar , “Prevention of Postpartum Stress Incontinence in Primigravidae With Increased Bladder Neck Mobility: A Randomised Controlled Trial of Antenatal Pelvic Floor Exercises,” BJOG: An International Journal of Obstetrics & Gynaecology 121, no. Suppl 7 (2014): 58–66. 25488090 10.1111/1471-0528.13213

[35] G. A. Digesu , N. Calandrini , A. Derpapas , P. Gallo , S. Ahmed , and V. Khullar , “Intraobserver and Interobserver Reliability of the Three‐Dimensional Ultrasound Imaging of Female Urethral Sphincter Using a Translabial Technique,” International Urogynecology Journal 23 (2012): 1063–1068. 22270730 10.1007/s00192-012-1669-3

[36] R. Karmarkar , A. Digesu , R. Fernando , and V. Khullar , “Urethral Sphincter Volume and Urodynamic Diagnosis,” International Urogynecology Journal 31 (2020): 2589–2594. 32613558 10.1007/s00192-020-04409-y

[37] G. A. Digesu , D. Robinson , L. Cardozo , and V. Khullar , “Three‐Dimensional Ultrasound of the Urethral Sphincter Predicts Continence Surgery Outcome,” Neurourology and Urodynamics 28 (2009): 90–94. 18726938 10.1002/nau.20566

[38] J. DeLancey , J. O. L. DeLancey , R. Kearney , Q. Chou , S. Speights , and S. Binno , “The Appearance of Levator Ani Muscle Abnormalities in Magnetic Resonance Images After Vaginal Delivery,” Obstetrics & Gynecology 101 (2003): 46–53. 12517644 10.1016/s0029-7844(02)02465-1 PMC1226664

[39] H. P. Dietz and V. Lanzarone , “Levator Trauma After Vaginal Delivery,” Obstetrics & Gynecology 106 (2005): 707–712. 16199625 10.1097/01.AOG.0000178779.62181.01

[40] K. L. Shek , J. Kruger , and H. P. Dietz , “The Effect of Pregnancy on Hiatal Dimensions and Urethral Mobility: An Observational Study,” International Urogynecology Journal 23 (2012): 1561–1567. 22584922 10.1007/s00192-012-1795-y

[41] G. A. van Veelen , K. J. Schweitzer , and C. H. van der Vaart , “Ultrasound Imaging of the Pelvic Floor: Changes in Anatomy During and After First Pregnancy,” Ultrasound in Obstetrics & Gynecology 44 (2014): 476–480. 24436146 10.1002/uog.13301

[42] P. Toozs‐Hobson , E. Edwards , A. Obloza , J. B. Toozs‐Hobson , and H. Egan , “Feasibility Study for the Value of Pelvic Floor Distension in Predicting Mode of Birth for Women Undergoing Vaginal Birth After Caesarean,” European Journal of Obstetrics & Gynecology and Reproductive Biology: X 10 (2021): 100126. 33855292 10.1016/j.eurox.2021.100126 PMC8024910

[43] A. Obloza and P. Toozs‐Hobson , “2D Uss of the Pelvic Floor in the 3rd Trimester Versus Mode of Delivery,” European Journal of Obstetrics & Gynecology and Reproductive Biology 230 (2018): 153–158. 30286365 10.1016/j.ejogrb.2018.09.040

[44] T. G. Gray , H. Vickers , P. Krishnaswamy , and S. Jha , “A Systematic Review of English language Patient‐Reported Outcome Measures for Use in Urogynaecology and Female Pelvic Medicine,” International Urogynecology Journal 32 (2021): 2033–2092, 10.1007/s00192-021-04810-1. 34037815

[45] L. T. Suzuki Zuchelo , I. M. Pinheiro Bezerra , A. T. Marcial Da Silva , et al., “Questionnaires to Evaluate Pelvic Floor Dysfunction in the Postpartum Period: A Systematic Review,” International Journal of Women's Health 10 (2018): 409–424, 10.2147/IJWH.S164266. PMC6087030 30123009

[46] M. A. Nijagal , S. Wissig , C. Stowell , et al., “Standardized Outcome Measures for Pregnancy and Childbirth, an ICHOM Proposal,” BMC Health Services Research 18 (2018): 953, 10.1186/s12913-018-3732-3. 30537958 PMC6290550

[47] A. L. Depla , B. Pluut , M. Lamain‐de Ruiter , et al., “PROMs and PREMs in Routine Perinatal Care: Mixed Methods Evaluation of Their Implementation Into Integrated Obstetric Care Networks,” Journal of Patient‐Reported Outcomes 7 (2023): 26, 10.1186/s41687-023-00568-w. 36894797 PMC9998006

[48] P. Sultan , N. Sharawi , L. Blake , et al., “Use of Patient‐Reported Outcome Measures to Assess Outpatient Postpartum Recovery: A Systematic Review,” JAMA Network Open 4 (2021): e2111600, 10.1001/jamanetworkopen.2021.11600. 34042993 PMC8160591

[49] M. Metz , B. Junginger , W. Henrich , and K. Baeßler , “Development and Validation of a Questionnaire for the Assessment of Pelvic Floor Disorders and Their Risk Factors During Pregnancy and Post Partum,” Geburtshilfe und Frauenheilkunde 77 (2017): 358–365, 10.1055/s-0043-102693. 28552999 PMC5406235

[50] K. Baessler , S. M. O'Neill , C. F. Maher , and D. Battistutta , “A Validated Self‐Administered Female Pelvic Floor Questionnaire,” International Urogynecology Journal 21 (2010): 163–172, https://doi.or/10.1007/s00192-009-0997-4. 19756341 10.1007/s00192-009-0997-4

[51] A. Fee , A. Uren , P. Abrams , et al. The Initial Development of the Perinatal Pelvic Health Self‐Assessment Questionnaire (2024), https://www.ics.org/2024/abstract/761.

[52] A. D. Uren , N. Cotterill , M. Pardoe , and P. Abrams , “The International Consultation on Incontinence Questionnaires (ICIQ): An Update on Status and Direction,” Neurourology and Urodynamics 39 (2020): 1889–1896, 10.1002/nau.24437. 32573011

[53] E. Jenabi , F. Shobeiri , S. M. M. Hazavehei , and G. Roshanaei , “Assessment of Questionnaire Measuring Quality of Life in Menopausal Women: A Systematic Review,” Oman Medical Journal 30, no. 3 (May 2015): 151–156, 10.5001/omj.2015.34. 26171119 PMC4459162

[54] Incontinence 7th Edition 2023. Editors Cardozo L, Rovner E, Wagg A, Wein A and Abrams P.

[55] M. Gyhagen , M. Bullarbo , T. Nielsen , and I. Milsom , “Prevalence and Risk Factors for Pelvic Organ Prolapse 20 Years After Childbirth: A National Cohort Study in Singleton Primiparae After Vaginal or Caesarean Delivery,” BJOG: An International Journal of Obstetrics & Gynaecology 120, no. 2 (January 2013): 152–160, Epub, 10.1111/1471-0528.12020. 23121158

[56] C. MacArthur , D. Wilson , P. Herbison , et al., “Faecal Incontinence Persisting After Childbirth: A 12 Year Longitudinal Study,” BJOG: An International Journal of Obstetrics & Gynaecology 120, no. 2 (2013): 169–179, 10.1111/1471-0528.12039. 23190303

[57] W. Y. Fu , H. Yuan , X. Q. Ye , D. Y. Shou , and W. Zhu , “Prediction of Postpartum Pelvic Floor Dysfunction With a Nomogram Model Based on Big Data Collected during Pregnancy,” Annals of Palliative Medicine 10, no. 2 (2021): 2143–2151, 10.21037/apm-21-166. 33549011

[58] I. Volløyhaug , S. Mørkved , Ø. Salvesen , and K. Å. Salvesen , “Forceps Delivery Is Associated With Increased Risk of Pelvic Organ Prolapse and Muscle Trauma: A Cross‐Sectional Study 16–24 Years After First Delivery,” Ultrasound in Obstetrics & Gynecology 46 (2015): 487–495, 10.1002/uog.14891. 25920322

[59] S. J. Snooks , M. Swash , M. M. Henry , and M. Setchell , “Risk Factors in Childbirth Causing Damage to the Pelvic Floor Innervation,” International Journal of Colorectal Disease 1, no. 1 (January 1986): 20–24, 10.1007/BF01648831. 3598309

[60] C. K. Mahoney , F. M. Reid , A. R. B. Smith , and J. E. Myers , “The Impact of Pregnancy and Childbirth on Pelvic Sensation: A Prospective Cohort Study,” Scientific Reports 13, no. 1 (2023): 1535, 10.1038/s41598-023-28323-7. 36707642 PMC9883213

[61] S. L. Jackson , D. Scholes , E. J. Boyko , L. Abraham , and S. D. Fihn , “Predictors of Urinary Incontinence in a Prospective Cohort of Postmenopausal Women,” Obstetrics & Gynecology 108, no. 4 (2006): 855–862, 10.1097/01.AOG.0000236446.17153.21. 17012446

[62] J. Al‐Mukhtar Othman , S. Åkervall , I. E. K. Nilsson , M. Molin , I. Milsom , and M. Gyhagen , “Fecal Incontinence in Nonpregnant Nulliparous Women Aged 25 to 64 Years‐A Randomly Selected National Cohort Prevalence Study,” American Journal of Obstetrics and Gynecology 226, no. 5 (May 2022): 706.e1–706.e23, 10.1016/j.ajog.2021.11.032. 34774822

[63] J. M. Lawrence , E. S. Lukacz , I. L. A. Liu , C. W. Nager , and K. M. Luber , “Pelvic Floor Disorders, Diabetes, and Obesity in Women,” Diabetes Care 30, no. 10 (2007): 2536–3541, 10.2337/dc07-0262. 17620443

[64] C. Rumeng , Z. Ya , C. Xirong , et al., “Work‐Related Factors Associated With the Pelvic Floor Dysfunction Among a Sample of Female Nurses in China,” Workplace Health & Safety 71, no. 6 (2023): 282–295, 10.1177/21650799231154282. 36988052

[65] J. E. Jelovsek , K. Chagin , M. Gyhagen , et al., “Predicting Risk of Pelvic Floor Disorders 12 and 20 Years After Delivery,” American Journal of Obstetrics and Gynecology 218, no. 2 (2018): 222.e1–222.e19, 10.1016/j.ajog.2017.10.014. 29056536

[66] D. Wilson , J. Dornan , I. Milsom , and R. Freeman , “Ur‐Choice: Can We Provide Mothers‐To‐Be With Information About the Risk of Future Pelvic Floor Dysfunction?,” International Urogynecology Journal 25, no. 11 (2014): 1449–1452, 10.1007/s00192-014-2376-z. 24740445

[67] Department of Health & Social Care , “Ministerial Foreword,” in Women's Health Strategy for England (Department of Health & Social Care, 2022), https://www.gov.uk/government/publications/womens-health-strategy-for-england/womens-health-strategy-for-england.

